# Epigenetic modulation of distal regulatory elements in oral squamous cell carcinoma (OSCC)

**DOI:** 10.1186/1868-7083-5-S1-S3

**Published:** 2013-08-19

**Authors:** Malgorzata Wiench, Songjoon Baek, Gordon Hager

**Affiliations:** 1 School of Dentistry, School of Cancer Sciences, The University of Birmingham, Birmingham, B15 2TT, UK; 2 Laboratory of Receptor Biology and Gene Expression, NCI, NIH, Bethesda, USA

## 

Epigenetic mechanisms have emerged as important contributors to cancer initiation and progression. DNA methylation of gene promoters has been extensively studied since the 1970s but the role of DNA methylation in the activity of distal regulatory elements (DREs) has only recently emerged. We have previously shown that tissue-specific differentially methylated regions overlap with DREs and that DNA methylation status correlates with their activity and the ability to bind transcription factors. We and others also demonstrated that such elements are dynamic and prone to demethylation. Our goal is to understand the role of DNA methylation and hydroxymethylation in the activity of DREs in cancer progression. OSCC, characterized by a double aetiology (exposure to carcinogens and HPV infection) and highly variable response to therapies, will be used as a model system.

Herein, we present the preliminary data of genome-wide identification of DREs in HPV-positive and HPV-negative OSCC cell lines using Digital DNaseI-Seq method and the application of this method in detection of chromosomal alterations – insertions and deletions. Data obtained at this stage will be used to establish a new methodological workflow for the 5mC and 5hmC analysis at DREs using capture array for fragment enrichment followed by third generation sequencing.

